# Excitotoxic superoxide production and neuronal death require both ionotropic and non-ionotropic NMDA receptor signaling

**DOI:** 10.1038/s41598-018-35725-5

**Published:** 2018-11-30

**Authors:** Angela M. Minnella, Jerry X. Zhao, Xiangning Jiang, Emil Jakobsen, Fuxin Lu, Long Wu, Jamel El-Benna, John A. Gray, Raymond A. Swanson

**Affiliations:** 1 Department of Neurology, University of California, San Francisco, San Francisco, CA 94122 USA; 2 San Francisco Veterans Affairs Medical Center, San Francisco, CA 94121 USA; 3 Center for Neuroscience and Department of Neurology, University of California Davis, Davis, CA 95618 USA; 4 Department of Pediatrics, University of California, San Francisco, San Francisco, CA 94143 USA; 5 Department of Drug Design and Pharmacology, Faculty of Health and Medical Sciences, University of Copenhagen, Copenhagen, 2100 Denmark; 6 INSERM-U1149, CNRS-ERL8252, Centre de Recherche sur l’Inflammation, Paris, France; 7 Université Paris Diderot, Sorbonne Paris Cité, Laboratoire d’Excellence Inflamex, Faculté de Médecine, Site Xavier Bichat, Paris, France

## Abstract

NMDA-type glutamate receptors (NMDAR) trigger superoxide production by neuronal NADPH oxidase-2 (NOX2), which if sustained leads to cell death. This process involves Ca^2+^ influx through NMDAR channels. By contrast, comparable Ca^2+^ influx by other routes does not induce NOX2 activation or cell death. This contrast has been attributed to site-specific effects of Ca^2+^ flux through NMDAR. Here we show instead that it stems from non-ionotropic signaling by NMDAR GluN2B subunits. To evaluate non-ionotropic effects, mouse cortical neurons were treated with NMDA together with 7-chlorokynurenate, L-689,560, or MK-801, which block Ca^2+^ influx through NMDAR channels but not NMDA binding. NMDA-induced superoxide formation was prevented by the channel blockers, restored by concurrent Ca^2+^ influx through ionomycin or voltage-gated calcium channels, and not induced by the Ca^2+^ influx in the absence of NMDAR ligand binding. Neurons expressing either GluN2B subunits or chimeric GluN2A/GluN2B C-terminus subunits exhibited NMDA-induced superoxide production, whereas neurons expressing chimeric GluN2B/GluN2A C-terminus subunits did not. Neuronal NOX2 activation requires phosphoinositide 3-kinase (PI3K), and NMDA binding to NMDAR increased PI3K association with NMDA GluN2B subunits independent of Ca^2+^ influx. These findings identify a non-ionotropic signaling pathway that links NMDAR to NOX2 activation through the C-terminus domain of GluN2B.

## Introduction

N-methyl-D-aspartate - type glutamate receptors (NMDAR) are expressed throughout the mammalian central nervous system. NMDAR are crucial for synaptic plasticity and learning, but can also mediate neuronal cell death. Most NMDAR are comprised of heteromeric assemblies of GluN1 and GluN2 subunits, both of which have C-terminal domains that extend into the neuronal cytoplasm. Agonist binding to glutamate sites on GluN2 subunits, in conjunction with co-agonist binding to glycine sites on GluN1 subunits, opens a receptor channel that is permeable to Ca^2+^ and other cations^1^. This ionotropic property is required for several downstream effects of NMDAR activation^2–4^. Though less widely recognized, NMDAR also have non-ionotropic signaling effects, i.e. signaling effects that are independent of receptor-gated ion flux^5–10^. These non-ionotropic signaling effects may be transmitted through ligand-induced conformational changes in the C-terminal cytoplasmic domains of GluN1 or GluN2 subunits^11^.

One of the downstream effects of neuronal NMDAR activation is production of superoxide^12^. Phsiological neuronal superoxide production has intercellular signaling functions^13,14^, but excessive neuronal superoxide production contributes to excitotoxic cell injury^15,16^. Superoxide produced in response to NMDAR stimulation is generated primarily by NADPH oxidase-2 (NOX2), and interventions that prevent NOX2 activation prevent excitotoxic cell death^17–22^. NMDA-induced NOX2 activation and neuronal death are both prevented by blocking Ca^2+^ influx through NMDAR^18,23,24^, indicating a requisite role for ionotropic, NMDAR-gated calcium influx. By contrast, Ca^2+^ influx of comparable magnitude through other routes does not trigger these events^24,25^. This contrast may be reconciled by the possibility that Ca^2+^ influx specifically through NMDAR has privileged access to local signaling pathways^25,26^. However, an alternative possibility is that NOX2 activation requires, in addition to Ca^2+^ influx, a non-ionotropic signaling effect induced by ligand binding to NMDAR.

Here we evaluated this alternative possibility using primary mouse cortical neuron cultures. Results of these studies show that NMDA - induced NOX2 activation and cell death requires, in addition to calcium influx, non-ionotropic signaling mediated by the C-terminal cytoplasmic domain of GluN2B subunits.

## Results

**Figure 1:**
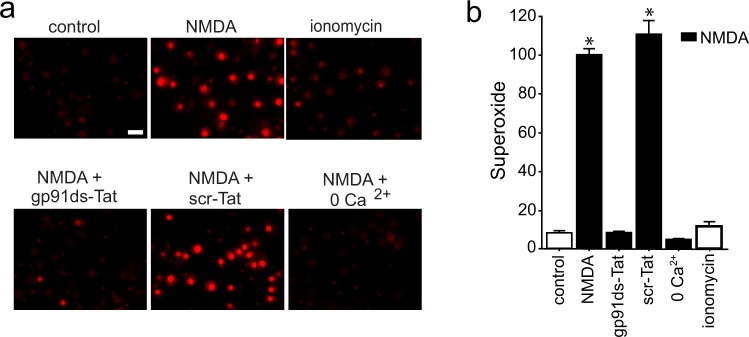
NMDA-induced superoxide production is Ca^2+^ -dependent, but not induced by Ca^2+^ influx alone. (**a**) Images show superoxide production, as measured by ethidium fluorescence (red), after incubation with NMDA (100 µM, 25 min). Scale bar = 20 µm. The NMDA-induced superoxide production is prevented by a Tat-conjugated peptide inhibitor of NOX2 (gp91ds-Tat, 3 µM) but not by a Tat-conjugated control peptide (scr-Tat). Superoxide production is also prevented by omitting Ca^2+^ from the medium. Superoxide production is not induced by ionomycin (3 µM). (**b**) Quantification of results shown in **a**, with results from each of n = 3 experiments expressed as percent of the NMDA-treated condition. Data are means + s.e.m.; *p < 0.01 vs control.

**Figure 2:**
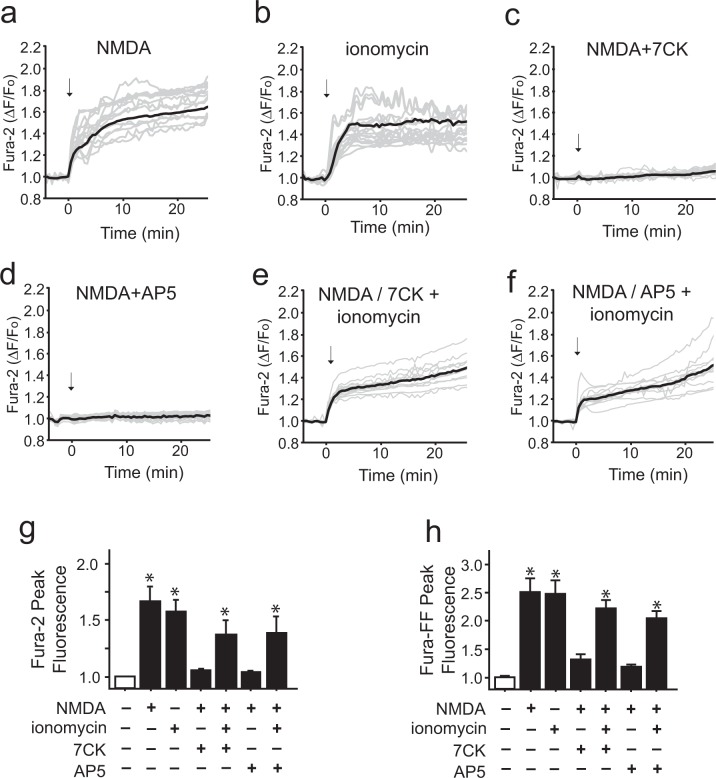
Ionomycin can mimic the intracellular Ca^2+^ elevation induced by NMDA. (**a**–**f**) Traces show mean changes in intracellular Ca^2+^ (black line) measured in individual neurons (gray lines) loaded with the high-affinity Ca^2+^ indicator Fura-2. Arrows mark addition of 100 µM NMDA or 3 µM ionomycin. The Ca^2+^ rise induced by NMDA is blocked by either 100 µM 7CK or 30 µM AP5 (**c**,**d**), and in the presence of these inhibitors the additional presence of 3 µM ionomycin reconstitutes the Ca^2+^ increase (**e**,**f**). (**g**,**h**) Panels show aggregate intracellular peak Ca^2+^ elevations as measured by the high affinity dye Fura-2 and low affinity dye Fura-FF, respectively. Note the mean Ca^2+^ elevation induced by 3 µM ionomycin does not exceed that induced by NMDA. Values are expressed relative to the mean values of the control condition in each of n = 4 experiments. Data are means + s.e.m.; *p < 0.05 vs control.

Using mouse cortical neuron cultures, we confirmed that NMDA - induced superoxide production was prevented by a peptide inhibitor of NADPH oxidase-2 (NOX2) (Fig. 1). This result is consistent with prior studies showing that NOX2 is the primary source of superoxide production during NMDAR activation (as reviewed in^16^). Superoxide production was also prevented by omitting Ca^2+^ from the medium, confirming that neuronal NOX2 activation requires Ca^2+^ influx. In contrast to NMDA, the Ca^2+^ ionophore ionomycin did not significantly increase superoxide production (Fig. 1) despite producing intracellular Ca^2+^ changes that were comparable in magnitude and kinetics to those produced by NMDA (Fig. 2, Suppl. Fig. S1).

**Figure 3:**
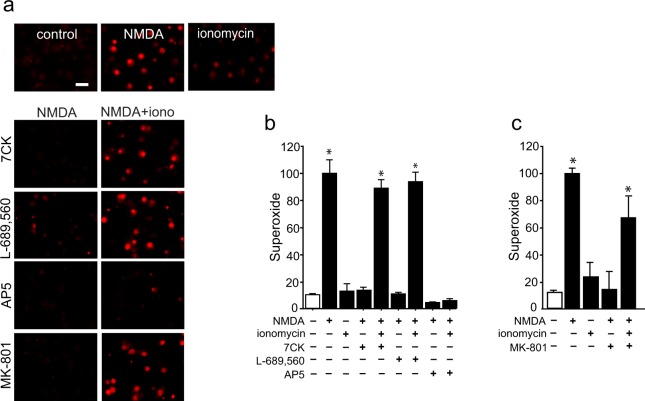
NMDA-induced superoxide production requires both Ca^2+^ influx and ligand binding. Images show superoxide production as measured by ethidium fluorescence (red). (**a**) NMDA-induced superoxide production, is prevented by 7CK (100 µM), L-689, 560 (1 µM), or MK801 (10 µM), which block NMDAR channel flux but not NMDA binding; and by 30 µM AP5, which blocks NMDA binding. The addition of ionomycin (3 µM) restores superoxide production in cultures treated with NMDA plus the channel inhibitors, but not in cultures treated with NMDA + AP5. (**b**,**c**) Quantification of results shown in (**a**) with results from each of n = 3 experiments expressed relative to the NMDA condition. Data are means + s.e.m.; *p < 0.05 vs control.

Given that NMDAR activation and ionomycin produced calcium elevations of similar rate and magnitude, but only NMDAR activation led to superoxide production, we asked whether superoxide production might require non-ionotropic signaling by NMDAR in addition to Ca^2+^ influx. To do this, we dissociated the non-ionotropic and ionotropic effects of NMDAR activation using a previously established pharmacological approach in which neurons are treated with the NMDAR glycine site antagonist 7-chlorokynurenate (7CK) to inhibit NMDAR channel opening without blocking NMDA binding^6–9,27^. Studies performed in this way confirmed that 7CK blocked NMDA-induced superoxide production (Fig. 3), as would be expected given its block of Ca^2+^ influx. When neurons treated with NMDA + 7CK were additionally treated with ionomycin to provide a Ca^2+^ influx, superoxide production was restored. Crucially, this result contrasts with the lack of superoxide production in neurons treated with ionomycin in the absence of NMDA. These results suggest that the signaling pathway linking NMDAR to superoxide production requires a non-ionotropic effect of ligand binding to NMDAR, in addition to Ca^2+^ influx.

The rate and magnitude of intracellular Ca^2+^ elevations induced by these treatments were assessed using the high-affinity calcium indicator Fura-2 (Fig. 2a–g). The rates of calcium elevations induced by NMDA and ionomycin were not significantly different, with half-maximal effects achieved within 4 minutes (Suppl. Fig. 1a) and near-maximal effects within 8 minutes (Fig. 2) in all conditions. The Fura-2 observations also confirmed that both 7CK and the competitive NMDAR inhibitor (2*R*)-amino-5-phosphonovaleric acid (AP5) effectively blocked Ca^2+^ elevations at the concentrations employed, and that the magnitude of Ca^2+^ elevation induced by the ionomycin + 7CK + NMDA condition did not exceed that induced by NMDA alone. We additionally evaluated the magnitude of Ca^2+^ elevation using the low affinity dye Fura-FF, to exclude the possibility that differences in maximal Ca^2+^ elevations over the duration of these studies might be masked by saturation of the high-affinity Fura-2 indicator. The results obtained with Fura-FF were in agreement with those obtained with Fura-2 (Fig. 2h).

7CK has been used in several prior studies to uncover non-ionotropic effects of NMDAR activation^6–9,27^, but nevertheless there is a possibility that off-target effects might affect the observations. Accordingly, the studies performed here with 7CK were also performed with a structurally distinct glycine site antagonist, L-689,560^28^ and with the open-channel blocker, MK-801. Like 7CK, L-689,560 and MK-801 prevented NMDA-dependent increases in superoxide production, and co-incubation with ionomycin reversed this effect (Fig. 3). The results obtained with 7CK, L-689,560, and MK-801 contrast with those obtained with the competitive NMDAR antagonist, AP5. AP5 blocks NMDA binding to GluN2 subunits of NMDAR and thus prevents non-ionotropic NMDA signaling, in addition to blocking NMDAR -mediated calcium influx. As expected, AP5 prevented NMDA-induced superoxide production and this effect was not reversed by co-incubation with ionomycin (Fig. 3).

**Figure 4:**
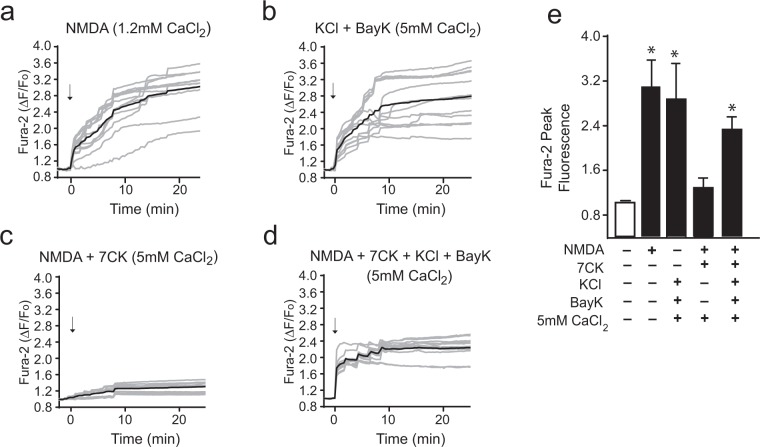
Activation of voltage-gated calcium channels can mimic the intracellular Ca^2+^ elevations induced by NMDA. (**a**–**d**) Traces show mean changes in intracellular Ca^2+^ (black line) measured in individual neurons (gray lines) loaded with the high-affinity Ca^2+^ indicator Fura-2. For clarity only every other obtained trace is shown. Arrows mark addition of either 100 µM NMDA or 50 mM KCl + 500 nM Bay K8644. Where indicated, the Ca^2+^ concentration in the medium was increased to 5 mM. The intracellular Ca^2+^ rise induced by NMDA is blocked by 100 µM 7CK (**c**), and in the presence of 7CK the addition of KCl + Bay K8644 reconstitutes the Ca^2+^ increase (**d**). (**e**) Panel shows the aggregate intracellular peak Ca^2+^ elevations as measured by Fura-2. Note the mean Ca^2+^ elevation induced by 50 mM KCl + Bay K8644 in 5 mM Ca^2+^ medium does not exceed that induced by NMDA. Values from each of n = 4 independent experiments are expressed relative to the control (no NMDA) condition. Data are means + s.e.m.; *p < 0.05 vs control.

**Figure 5:**
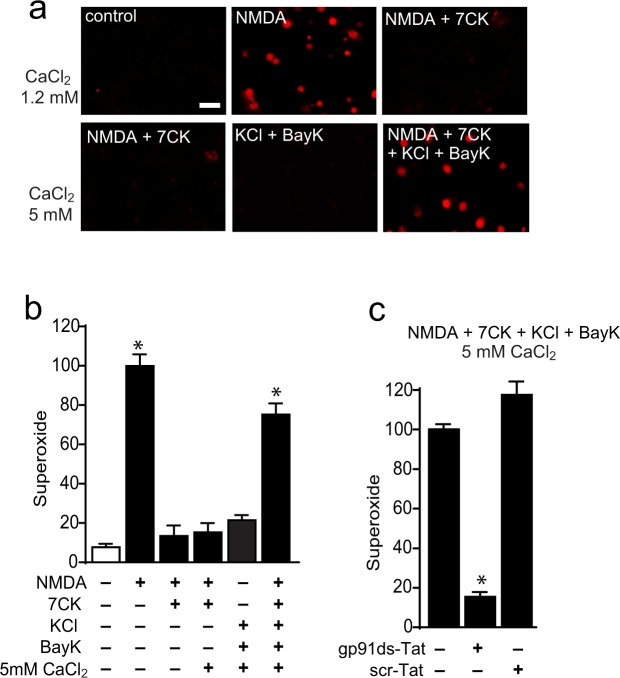
NMDA-induced superoxide production requires both Ca^2+^ influx and ligand binding. Images show superoxide production as measured by ethidium fluorescence (red). (**a**) NMDA-induced superoxide production is prevented by the NMDAR glycine site antagonists 7CK, which blocks NMDAR channel opening but not NMDA binding. The addition of 500 nM Bay K8644 in medium containing 50 mM KCl and 5 mM CaCl_2_ restores superoxide production in cultures treated with NMDA + 7CK. Scale bar = 10 µm (**b**) Quantification of results shown in **a**, with results from each of n = 4 experiments expressed relative to the NMDA treated condition. *p < 0.01 vs control. (**c**) Superoxide production induced by the NMDA + 7CK + KCl + Bay K8644 + 5 mM CaCl_2_ treatment condition is blocked by the NOX2 inhibitory peptide gp91 ds-Tat (3 µM), but not the control peptide scr-Tat. Data are means + s.e.m; n = 3, *p < 0.01.

We also considered the possibility that ionomycin might have off-target effects that could influence the observations. We therefore also used a second way to induce Ca^2+^ influx, activation of voltage-gated calcium channels. This was accomplished as previously described^26,29^, using elevated KCl (50 mM) to cause depolarization, 1,4-dihydro-2,6-dimethyl-5-nitro-4-(2-[trifluoromethyl]phenyl)pyridine-3-carboxylic acid methyl ester (Bay K8644, 500 nM) to prevent channel inactivation, and an elevated medium Ca^2+^ content (5 mM). These conditions produced a calcium influx of similar rate and magnitude to that achieved with NMDA (Fig. 4, Suppl. Fig. 1b). As shown in Fig. 5, results of studies using this method to induce Ca^2+^ elevations gave results parallel to those observed with ionomycin. The intracellular Ca^2+^ rise induced by KCl + Bay K8644 + 5 mM calcium did not itself induce superoxide production, nor did ligand binding to NMDAR in the absence of calcium influx (NMDA + 7CK). However, the calcium influx induced by KCl + Bay K8644 + 5 mM calcium when combined with ligand binding (NMDA + 7CK) induced a robust superoxide production (Fig. 5a,b). This superoxide production was attributable to NOX2 activation, as it was completely negated by the NOX2 inhibitory peptide gp91ds-Tat (Fig. 5c).

**Figure 6:**
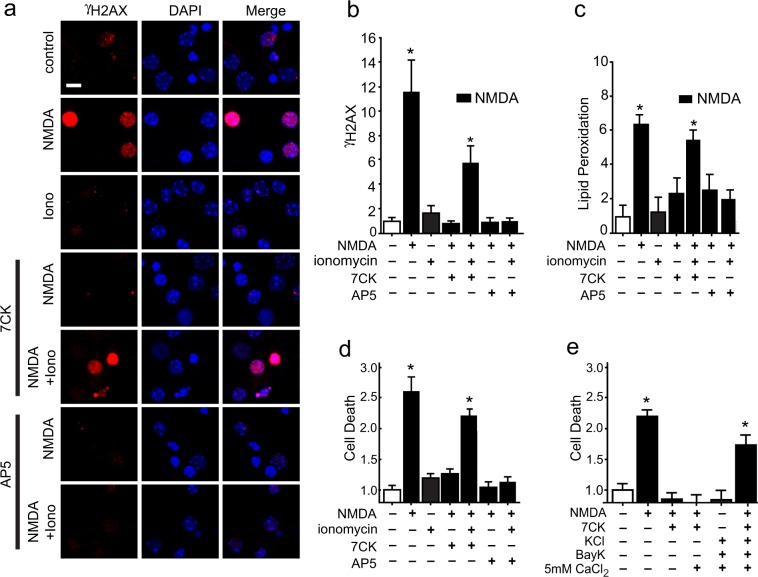
NMDA-induced DNA damage, lipid peroxidation, and cell death require both Ca^2+^ influx and ligand binding. (**a**) Double stranded DNA breaks are visualized by foci of γH2Ax immunoreactivity (red) after incubation with either NMDA or NMDA + 7CK + ionomycin, but not after incubation with NMDA + AP5 + ionomycin. Scale bar = 5 µm. (**b**) Quantification of results shown in **a**. (**c**) Increased lipid peroxidation after incubation with NMDA or NMDA + 7CK + ionomycin, but not after incubation with NMDA + AP5 + ionomycin. (**d**) Increased cell death after incubation with NMDA or NMDA + 7CK + ionomycin, but not after incubation with NMDA + AP5 + ionomycin or with ionomycin in the absence of NMDA. (**e**) Increased cell death after incubation with NMDA or NMDA + 7CK + voltage-gated calcium channel activation (Bay K8644 in 50 mM KCl and 5 mM CaCl_2_), but not after activation of voltage-gated calcium channels in the absence of NMDA. Treatment conditions are as in Figs 3 and 5. Data are means + s.e.m., expressed as fold increase over control. Cell death in the control groups was 21 ± 8%. n = 3–5; *p < 0.05 vs control.

We confirmed the effects of non-ionotropic NMDAR signaling on superoxide production with two additional markers of oxidative stress, DNA damage and lipid peroxidation, and by assessing cell death. DNA strand breaks (as identified by foci of γH2AX accumulation) and lipid peroxidation were observed in neurons treated with NMDA alone or with NMDA + 7CK + ionomycin, but not in neurons treated with NMDA + AP5 + ionomycin (Fig. 6a–c), indicating a requirement for agonist binding to NMDAR in addition to Ca^2+^ influx. Assessment of neuronal death at 24 hours after NMDA exposure revealed the same pattern, using either ionomycin or KCl + Bay K8644 + 5 mM CaCl_2_ to provide calcium influx during NMDAR ligand binding (Fig. 6d,e).

**Figure 7:**
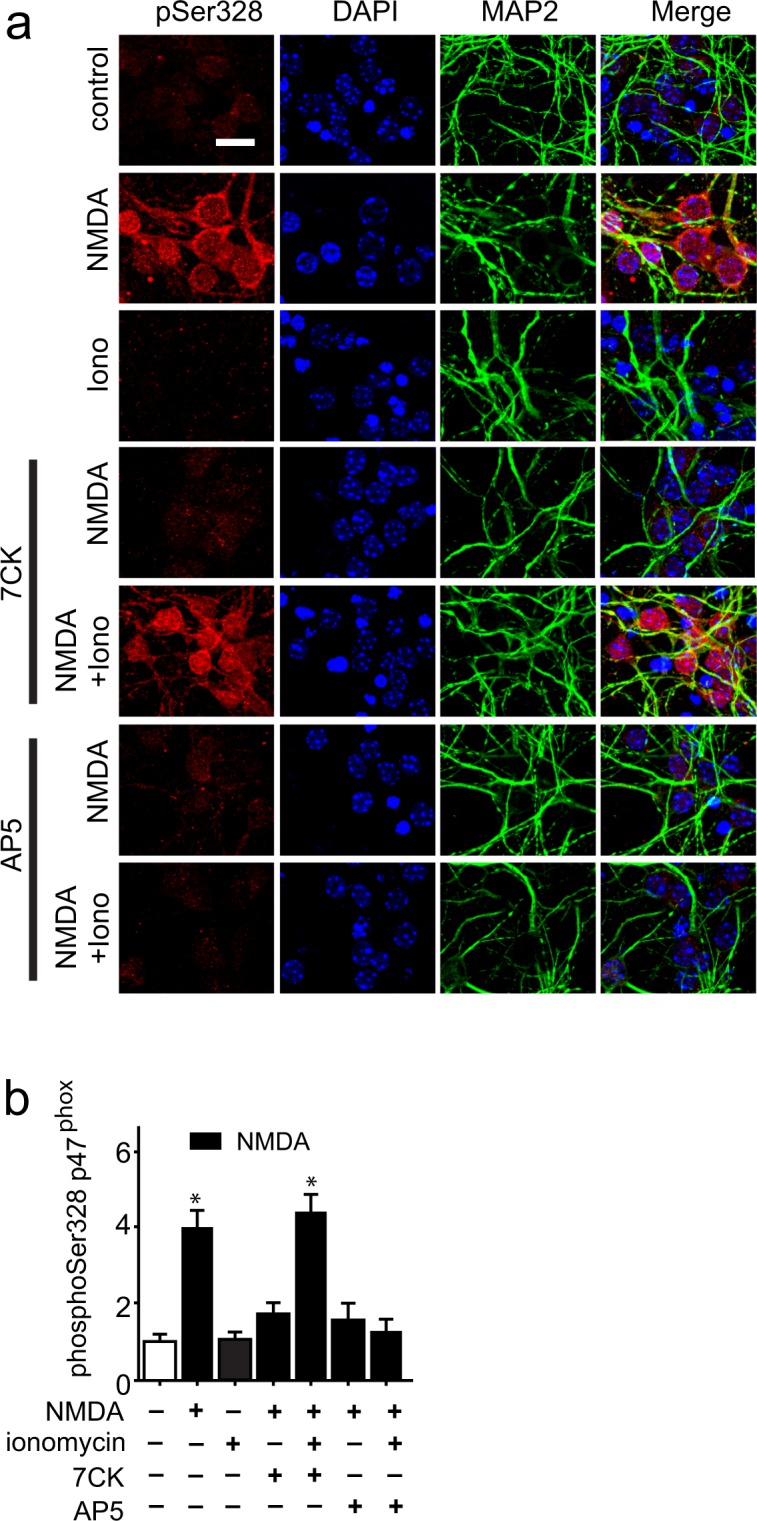
Phosphorylation of NADPH oxidase p47^phox^ requires both Ca^2+^ influx and ligand binding. Scale bar = 5 µm. (**a**) Increased phosphorylation of p47^phox^ at Ser328 occurs after incubation with NMDA or NMDA + 7CK + ionomycin, but not after incubation with NMDA + AP5 + ionomycin or with ionomycin in the absence of NMDA. Conditions are as in Fig. 3. (**b**) Quantification of results shown in (**a**), with data in each of n = 3 experiments normalized to the control condition; *p < 0.05 vs control.

The assembly of an activated NOX2 complex requires phosphorylation of the NOX2 p47^phox^ subunit at serine 328^24,30^. Immunostaining for phospho-Ser328-p47^phox^ confirmed that NMDAR activation induced phosphorylation of this residue (Fig. 7). Importantly, these studies also showed that the p47^phox^ phosphorylation required a non-ionotropic effect of NMDAR stimulation: p47^phox^ became phosphorylated in cultures treated with NMDA + 7CK + ionomycin (which prevents ion flux through the NMDAR but not ligand binding), but not in cultures treated with NMDA + AP5 + ionomycin (which prevents NMDA binding).

**Figure 8:**
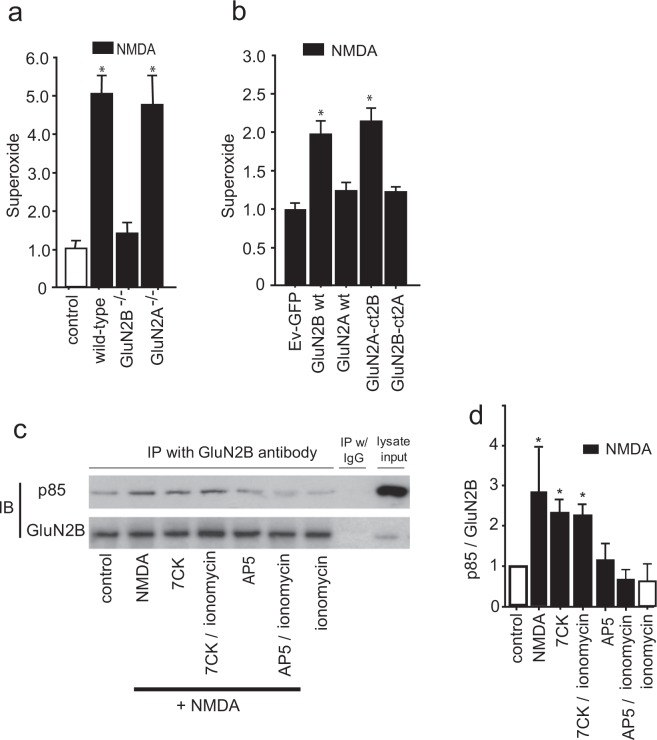
NMDA-induced superoxide production is mediated through the C-terminal cytoplasmic domain of GluN2B and is associated with PI3K binding. (**a**) Selective deletion of GluN2B but not GluN2A subunits prevents superoxide formation induced by NMDA. Cultures were treated with NMDA as in Fig. 3, with results from each of n = 3 experiments expressed relative to the NMDA treated condition; *p < 0.05 vs control. (**b**) In neurons lacking both endogenous GluN2A and GluN2B, NMDA-induced superoxide production was observed in cultures transfected with either the GluN2B subunit (GluN2B wt) or the GluN2A subunit fused to a GluN2B C-terminal domain (GluN2A-ct2B), but not in cultures transfected with GluN2A wt or GluN2B-ct2A. Results from each of 3 experiments are expressed relative to signal obtained in cultures transfected with the empty vector GFP (Ev-GFP). The reduced magnitude of the NMDA effect in the GluN2B - transfected neurons (**b**) relative to that observed in Wt neurons (**a**) is consistent with the transfection efficiency of approximately 10% achieved with each of the GluN2 constructs. Data are means + s.e.m.; *p < 0.05 v Ev-GFP. (**c**) Immunoblots (IB) of PI3K p85 and GluN2B in lysates immunoprecipitated (IP) with antibody to GluN2B. IP with normal mouse IgG served as negative control. The p85 band is at ~90 kD and the GluN2B band at ~200 kD. An aliquot of the cell lysate was used as an input control to verify the presence of detected protein in the original samples. Full-length blots are presented in Supplementary Fig. S4. (**d**) Quantified results of p85 binding to GluN2B normalized to the control values; n = 3; *p < 0.05 vs control.

Evidence suggests that NMDAR that contain GluN2B subunits are preferentially involved in superoxide production and excitotoxic cell death^24,31,32^. Here we prepared neurons deficient in GluN2B, GluN2A, or both subunit types using Cre-induced excision of their corresponding floxed loci^33^. NMDA-induced superoxide production was robust and comparable in Wt and GluN2A deficient neurons, but negligible in GluN2B deficient neurons (Fig. 8a), suggesting signaling through GluN2B but not GluN2A. However, an alternative explanation for this result is low-level GluN2A expression in the cultures, as GluN2A expression is low embryonically and increases with age^34^. Accordingly, we also evaluated neurons that were rendered deficient in native GluN2A and GluN2B and transfected to selectively express either GluN2A or GluN2B only. This approach was also used to selectively express chimeric GluN2A containing C-terminal GluN2B, or chimeric GluN2B containing C-terminal GluN2A. In neurons lacking both GluN2A and GluN2B, cells transfected with GluN2B but not GluN2A exhibited superoxide production in response to NMDA (Fig. 8b), thus supporting a specific role for GluN2B in this process. Importantly, GluN2A/GluN2B deficient neurons that were transfected with a chimeric GluN2A construct containing the GluN2B C-terminus (GluN2A-ct2B) also exhibited superoxide production, while those transfected with chimeric GluN2B subunits containing the GluN2A C-terminus (GluN2B-ct2A) did not (Fig. 8b). Prior studies have demonstrated efficient transfection-mediated expression of each of these constructs in immature neuron cultures^35–38^, and electrophysiological characterization of these constructs expressed in brain slices confirmed that their intrinsic conductances are very similar (Suppl. Fig. S2). Together, these observations support the importance of the GluN2B C-terminus in NMDAR-induced NOX2 activation, and argue against the possibility that the inability of GluN2A subunits to induce NOX2 activation is because of reduced GluN2A expression or current flux.

The signaling pathway linking NMDAR activation to NOX2 activation requires PI3K activation, which in turn induces the phosphorylation of p47^phox^ by the atypical (PI3K-activated) protein kinase C, PKCzeta^24^. NMDA-induced PI3K activation involves binding of the p85 regulatory subunit of PI3K to GluN2B, either directly or via an adaptor protein^39–41^. To determine whether this binding is induced by non-ionotropic NMDAR signaling, we immunoprecipitated GluN2B and evaluated for co-precipitation of p85. Results of these studies showed an NMDA-induced association of p85 with GluN2B even in the presence of 7CK, indicating a non-ionotropic mechanism. As expected, this association was blocked by AP5 and not induced by ionomycin (Fig. 8c,d).

The factors determining p85 binding to GluN2B are poorly understood. However, several lines of evidence indicate that phosphorylation at GluN2B Tyr1472 is required for NMDA-induced NOX2 activation^42,43^, and that phosphorylation at GluN2B Tyr1336 promotes p85 binding^40^. Phosphorylation at these sites is mediated by Src kinases, predominately Src and Fyn, which when activated are themselves phosphorylated at Tyr416. We evaluated phosphorylation at each these sites by preparing immunoblots from cultures treated for 5 minutes with NMDA or NMDA + 7CK. These blots did not, however, reveal any significant NMDA-induced changes in the phosphorylation status of these sites (Suppl. Fig. S3). The 5 -minute incubation period was used to avoid GluN2B cleavage during NMDA incubation^44^. An additional set of studies using 30-minute incubation intervals also revealed no significant phosphorylation changes.

## Discussion

Ca^2+^ elevations mediated by NMDAR activation lead to excitotoxic neuronal death, whereas comparable Ca^2+^ elevations mediated by other Ca^2+^ entry routes do not^25,26^. The reason for this difference has not been established, but it is widely thought to stem from a privileged access to local signaling pathways by Ca^2+^ influx through NMDAR. The present findings suggest instead that this difference is attributable to non-ionotropic signaling induced by agonist binding to GluN2B - containing NMDAR. They demonstrate that Ca^2+^ influx produced by either ionomycin or voltage-gated calcium channels fails to induce superoxide formation or neuronal death unless there is a concomitant binding of ligand to NMDAR. These findings further indicate that the GluN2B C-terminal domain mediates this non-ionotropic effect of NMDAR activation. Prior studies have shown that PI3K activation is required for neuronal NOX2 activation^24^, and the present results also show that agonist binding to NMDAR (in the absence of Ca^2+^ influx) is sufficient to induce association of the PI3K p85 regulatory subunit to GluN2B.

Our findings add to previously identified roles for non-ionotropic NMDAR signaling^45^. This signaling mechanism was first described by Westbrook and colleagues, who found that agonist binding independent of ion flux could downregulate current flux through NMDAR through a process involving de-phosphorylation of GluN1 tyrosine residues^5^. Non-ionotropic effects of NMDAR were subsequently shown to mediate long-term depression, p38 phosphorylation^6,7^, and dendritic spine shrinkage^9^. A synergistic interaction between NMDAR and mGlu5 receptors on gene expression was likewise found to require NMDAR agonist binding but not current flux^46^. In addition to glutamate binding, glycine binding to NMDA receptors on its GluN2A subunit has been shown to have a non-ionotropic effect that promotes cell survival when ionic flux is blocked^47^. None of these reports identify the molecular mechanism by which ligand binding mediates these effects, but a study using fluorescence resonance energy transfer (FRET) demonstrated that agonist binding to NMDAR, without ion flow, can drive rapid and reversible movement of C-terminus cytoplasmic domains^11,48^, suggesting that conformational changes in these domains enable subsequent downstream signaling.

Superoxide has a variety of intercellular signaling roles both in brain and other tissues^13,14,49,50^. However, superoxide and its downstream reactive oxygen metabolites (including hydrogen peroxide, hydroxyl radical, peroxynitrite and others) can also contribute to pathogenesis of stroke and neurodegenerative disorders^15,16,51,52^. Superoxide can be produced by several mechanisms, but in the present studies the complete suppression of the superoxide ethidium signal with a peptide inhibitor of NOX2 established NOX2 as the primary source of NMDA-induced superoxide production. This is consistent with several prior reports in which suppression of NOX2 activity by pharmacological or genetic approaches blocked the superoxide formation, lipid oxidation, DNA damage, and cell death resulting from NMDAR activation^16,18,20,53^. Elevations in intracellular Ca^2+^ to levels substantially above those induced by 100 µM NMDA can, however, induce neuronal superoxide production from a source that is insensitive to NOX2 inhibitors, presumably mitochondria^24,29^. The ionomycin and voltage-gated calcium activation conditions used in the present studies were therefore titrated to ensure that neither the rates nor magnitude of Ca^2+^ elevation exceeded those induced by NMDA. Our measures of intracellular Ca^2+^ elevations were made in neuronal soma, which could underestimate (or overestimate) elevations that occur in subdomains such as dendritic processes or synaptic boutons. That, however, would not affect the conclusion that the Ca^2+^ elevations alone do not induce superoxide production or cell death in the absence of ligand binding to NMDAR.

The use of primary neuron cultures in these studies facilitated genetic and pharmacological manipulations and the measurements of Ca^2+^ elevations and superoxide production; however, cultured neurons do not fully reflect behavior of neurons *in vivo*. In particular, they exhibit reduced expression of the GluN2A NMDAR subunit during the first two weeks *in vitro*^34^. This limitation can be circumvented by transfecting the neurons with native or chimeric GluN2 constructs^35–38^, as performed in the present study. Here, NMDA induced superoxide production in neurons that selectively expressed GluN2B (but not GluN2A) and in neurons that selectively expressed chimeric GluN2A containing C-terminal GluN2B (but not GluN2B containing C-terminal GluN2A), indicating that the GluN2B C-terminal domain is required. This is an important aspect of our results, because GluN2B - containing NMDAR have previously been shown to have a dominant role in both neuronal NOX2 - mediated superoxide production and excitotoxic cell death^24,36,54^.

A second limitation to this study is that it does not identify the non-ionotropic signal transduction mechanism at the molecular level. Our results do point to a potential mechanism by showing that agonist binding to NMDAR increases the association between GluN2B and the p85 regulatory subunit of PI3K. This is significant because PI3K activity is required to activate protein kinase C zeta, which in turn phosphorylates and activates the NOX2 p47^phox^ subunit^24^. As prior studies using FRET have shown agonist – induced conformational changes in NMDAR C-terminus domain^11,48^, this observation suggests that p85 binding to GluN2B may be enabled by a conformation change triggered by NMDA binding to GluN2B. Although our studies did not identify changes in GluN2B phosphorylation state associated with p85 binding, they do not exclude phosphorylation that may occur selectively in the synaptic, membrane-bound pool of NMDAR or at extrasynaptic sites, or other types of post-translational modifications. It is also possible that a conformational change alone is sufficient to permit p85 binding, without associated changes in phosphorylation, acetylation, or other modifications.

Non-ionotropic signaling provides an additional layer of complexity to NMDAR physiology. A study published during preparation of this manuscript describes a neuroprotective effect of non-ionotropic NMDAR in brain ischemia, through a mechanism involving AMPA receptor function^55^. Another recent study identifies a role for non-ionotropic NMDA signaling in the cytotoxic opening of pannexin channels under excitotoxic conditions, and places irreversible neuronal injury downstream of pannexin channel opening^10^. It will be thus be important to determine whether the signaling pathway identified here in neuronal cultures is likewise functioning in intact brain, and whether it contributes to pannexin channel opening under excitotoxic conditions.

The capacity for both ionotropic and non-ionotropic NMDAR signaling may allow NMDA receptor complexes to initiate different signaling events depending upon subunit composition, agonist and co-agonist availability, and intracellular Ca^2+^ levels. Given that intracellular Ca^2+^ dynamics are influenced by the aggregate effect of local NMDAR activation, this property may also provide a mechanism whereby the non-ionotropic effect of agonist binding to single NMDAR can be influenced by concomitant ionotropic activity of neighboring NMDAR.

## Materials and Methods

Studies were performed in accordance with protocols approved by the University of California, San Francisco and San Francisco Veterans Affairs Medical Center animal studies subcommittees. Cell culture reagents were obtained from Mediatech, and all other reagents were obtained from Sigma-Aldrich except where noted. Data analyses for all studies were performed by observers blinded to the experimental conditions.

### Cortical cultures

Mixed neuron-astrocyte cultures were prepared from the cortices of embryonic day 16–18 mice and plated in 24-well culture plates or poly-D-lysine coated glass coverslips^18^. After 4 days in culture, 1.2 µM cytosine arabinoside was added to culture media to suppress astrocyte proliferation. The cells were subsequently maintained in NeuroBasal medium (Gibco) containing 5% FBS, 5 mM glucose, and used at day 10–15 *in vitro*.

### Experimental conditions

The culture medium was replaced with a low-magnesium balanced salt solution (BSS): 137 mM NaCl_2_, 1.2 mM CaCl_2_, 0.4 mM MgSO_4_, 5.3 mM KCl, 0.4 mM NaH_2_PO_4_, 0.3 mM Na_2_HPO_4_, 5 mM glucose, and 10 mM 1,4-piperazinediethanesulfonate (PIPES) buffer, pH 7.3. Where a “high calcium” medium was used, the CaCl_2_ concentration was increased to 5 mM and NaCl concentration was reduced to 133 mM. Experiments were initiated by the addition of either N-methyl D-aspartate (NMDA), ionomycin, or KCl plus Bay K8644 from concentrated stocks. Where used, the NMDA receptor blockers 7CK, AP5, MK-801, and L-689,560 were added 10 minutes prior to NMDA.

### Intracellular calcium imaging

Neurons were loaded for 30 minutes with either 4 µM Fura-2 AM or Fura-FF AM (Molecular Probes) and washed once with BSS prior to imaging. Images were acquired at 20 second intervals, using excitation that alternated between 340 nm and 380 nm (emission > 510 nm). Raw fluorescence was normalized to baseline levels prior to stimulus. Calcium elevations were then expressed as change in the ratio of 340 nm/380 nm fluorescence. Fura-FF measures were all well below saturation^24^. Maximal values and time to half-maximal values were calculated over the interval of 0–25 minutes after addition of test compounds to the media, using the normalized data obtained from more than 10 neurons from each of n = 3 independent experiments.

### Superoxide imaging

Five µM dihydroethidium (Invitrogen) was added to cultures 10 minutes prior to the addition of the test compounds (NMDA, ionomycin, or Bay K8644) and maintained throughout the experiment. The cultures were photographed after 25 minutes incubation with a fluorescence microscope (Axiovert 40 CFL, Zeiss) using 510–550 nm excitation and >580 nm emission, and with transmitted light phase contrast. The fluorescence of oxidized ethidium species (Eth) was measured in 3 randomly chosen optical fields from each culture well and calculated as mean Eth fluorescence [area (pixels) X intensity (arbitrary units)] normalized to the cell number counted in the phase contrast images of each optical field. Measurements were made in 4 wells per experiment, with >100 neurons per well for a total of >500 neurons per “n”. Superoxide (or a superoxide metabolite) was confirmed as the reactive oxidant by negation of Eth formation in the studies where NOX2 activity was blocked.

### Live cell lipid peroxidation

Image-iT lipid peroxidation kit was purchased from Molecular Probes, and labeling and detection methods were followed as described in the protocol. Briefly, cells were treated with drugs as described above, and the lipid peroxidation sensor (10 µM) was added to each well. After 20 minutes, the cells were washed twice with control BSS and images were prepared from 3 randomly selected regions in each well to evaluate fluorescence at 590 nm to 510 nm. The ratio of signal intensities was calculated as a measure of lipid peroxidation. Measurements were made in 4 wells per experiment, with >100 neurons per well, for a total of 400–500 neurons per “n”.

### GluN2 subunit deletion and C-terminal domain swaps

Primary neuron cultures were prepared as described using *Grin2a*^fl/fl^ (GluN2A) or *Grin2b*^fl/fl^ (GluN2B) mice^33,56^. On culture day 2, the cultures were infected with rAAV1-Cre:BFP viral stock (~1 × 10^12^ vg/ml), expressing a nuclear targeted Cre:BFP fusion protein. Experiments were initiated after 11 days in culture. For C-terminal domain swap studies, cultures were prepared from double-floxed *Grin2a*^*fl/fl*^*Grin2b*^*fl/fl*^ (GluN2A/GluN2B) mice. Cultures were infected on day 2 in culture with rAAV1-Cre:BFP to eliminate expression of the floxed genes, and lipofectamine transfected on culture day 6 with either pCAGGS-GFP or pCAGGS-GluN2X-IRES-GFP (where X is wild-type or chimeric GluN2A or GluN2B). The C-terminal domains were swapped at E838 of GluN2A and E839 of GluN2B. Transfection efficiency was assessed as the fraction of GFP-positive cells in each well, and showed no significant differences among the constructs used.

### Immunostaining

Formaldehyde-fixed cultures were immunostained as described previously^18^ using mouse antibody to microtubule-associated protein 2 (MAP2, Chemicon, 1:500) or rabbit antibody to anti-Ser129-Histone H2AX (1:500, Cell Signaling Technology). Antibodies to phospho-p47^phox^ (Ser 328) were generated and characterized previously^57^. Antibody binding was visualized with Alexa Fluor 488-conjugated anti-mouse IgG or Texas Red 594-conjugated anti-rabbit IgG (Invitrogen). Images were obtained using confocal fluorescence microscopy using step-wise 1 µm z-stack sampling. In some cases cell nuclei were also identified for fluorescence imaging by incubation with 1 µM 4′, 6-diamidino-2-phenylindole (DAPI) after antibody removal. Neurons were analyzed in 3 randomly chosen optical fields from each of 4 coverslips per experiment, with >100 neurons per coverslip, for a total of 400–500 neurons per “n”. Staining was quantified as fluorescence [area (pixels) ×intensity (arbitrary units)]/neuronal surface area (pixels with MAP2 immunoreactivity).

### Immunoblots

Cultures were treated under the designated conditions for either 5 or 30 minutes at 37 °C, then harvested in RIPA lysis buffer containing protease and phosphatase inhibitors (ThermoFisher Scientific). Antibodies to the following epitopes were used: GluN2B N-terminus (MyBioSource, San Diego, CA, # MBS800075); phospho-Y1336 GluN2B (PhosphoSolutions, Aurora, CO, #p1516–1336); phospho-Y1472 GluN2B (Cell Signaling Technology, Boston, MA, #4208); pY416 Src (Cell Signaling Technology, #2101) and ß-actin (Santa Cruz Biotechnology, sc-47778). Appropriate secondary horseradish peroxidase-conjugated antibodies were used, and signal was visualized with enhanced chemiluminescence. Image J was used to measure the optical densities (OD, area × intensity) of the protein bands.

### Immunoprecipitation (IP)

Cultures were treated under the designated conditions for 5 minutes at 37 °C, then harvested in RIPA lysis buffer containing protease and phosphatase inhibitors. Samples were diluted with IP buffer (PBS containing 1% NP40 and protease and phosphatase inhibitors) and pre-cleared with Protein G beads (Invitrogen). IP was performed overnight at 4 °C with 4 μg mouse anti-GluN2B (MyBioSource, #MBS800075) or 4 μg normal mouse IgG (IgG2b, k isotype, BioLegend, #401202) as a negative control, then incubated with Protein G beads for 2 hr at 4 °C with rotation. After washing and centrifugation, the beads were boiled for 10 min in reducing sample buffer (Invitrogen). The samples were applied to SDS-PAGE electrophoresis and transferred to PVDF membranes. The membranes were incubated with rabbit GluN2B antibody (Cell Signaling Technology, #4212) or rabbit p85 antibody (Cell Signaling Technology, #4257), and then with goat anti-rabbit HRP-conjugated secondary antibodies. Immunoreactive proteins were visualized with enhanced chemiluminescence. The binding of p85 with GluN2B was expressed as the OD ratio of co-immunoprecipitated p85 with GluN2B.

### Cell death

Cultures were incubated under the designated experimental conditions for 30 minutes at 37 °C, then transferred to fresh BSS and incubated for an additional 24 hours. All incubations were in 0% CO_2_ (room air). At 24 hours calcein green-AM (5 µM, Molecular probes) was added to identify living cells, and Hoechst 33258 (1 µM, Molecular probes) to identify all cell nuclei. Analysis at the 24 hour time point was done to preclude the possibility of calcein leakage from viable cells through transiently opened pores. Photographs were taken of 3 randomly determined fields of each culture well, and cell death was quantified by counting the number of cells labeled by Hoechst 33258 but not calcein green^27^. Cells were counted in 3 fields from a minimum of 4 wells per plate in each independent experiment.

### Statistical analyses

In all cases, the ‘n’ values denote the number of independent experiments, each using neurons prepared from different mice. Each independent experiment contained triplicate culture wells or coverslips of each study condition, with measurements obtained from at least 200 neurons in each culture well or 10 neurons on each coverslip. Data are expressed as means ± s.e.m. and assessed using one-way ANOVA followed by the Tukey-Kramer test where multiple groups are compared against one another, or Dunnett’s test where multiple groups are compared against a common control group.

## Electronic supplementary material

Supplemental Figures

## Data Availability

Full length western blots corresponding to the cropped images presented in the figures are provided as Supplementary Fig. 4. All datasets generated during this study are available from the corresponding author upon request.
