# Supplementary material for: Excitotoxic superoxide production and neuronal death require both ionotropic and non-ionotropic NMDA receptor signaling
Source: Sci Rep. 2018 Nov 30;8:17522. doi: 10.1038/s41598-018-35725-5 (PMC6269523; doi:10.1038/s41598-018-35725-5)
Supplement: Supplementary file 1 — Supplemental Figures [file 41598_2018_35725_MOESM1_ESM.pdf]

SUPPLEMENTAL FIGURES

Excitotoxic superoxide production and neuronal death require both ionotropic and non-ionotropic NMDA receptor signaling

Angela M. Minnella, Jerry X. Zhao, Xiangning Jiang, Emil Jakobsen, Fuxin Lu, Long Wu, Jamel El-Benna, John A. Gray, Raymond A. Swanson

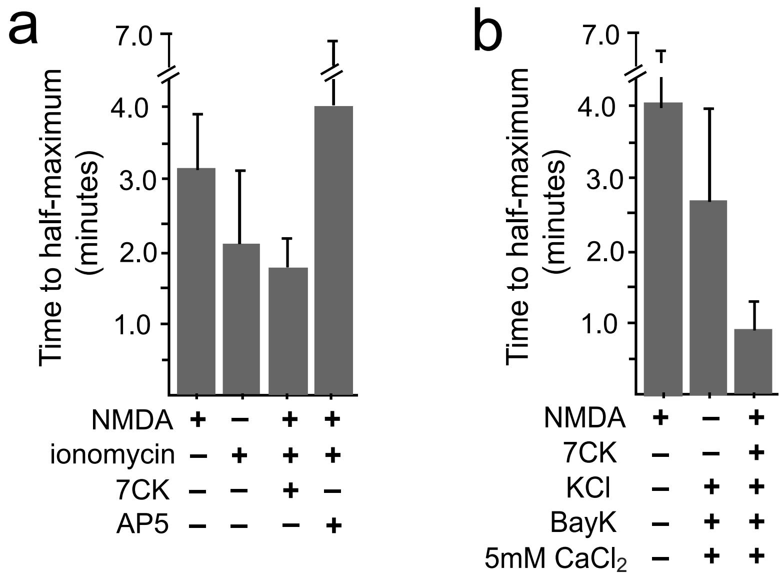

Supplementary Figure S1. Intracellular calcium elevation rates. (a) Time required for the Fura-2 signal shown in Figure 2 to reach the half maximal value observed over the 25 minute observation period. (b) Time required for the Fura-2 signal shown in Figure 4 to reach the half maximal value observed over the 25 minute observation period. Data are means ± s.e.m. from > 30 neurons in 4 experiments (n =4).

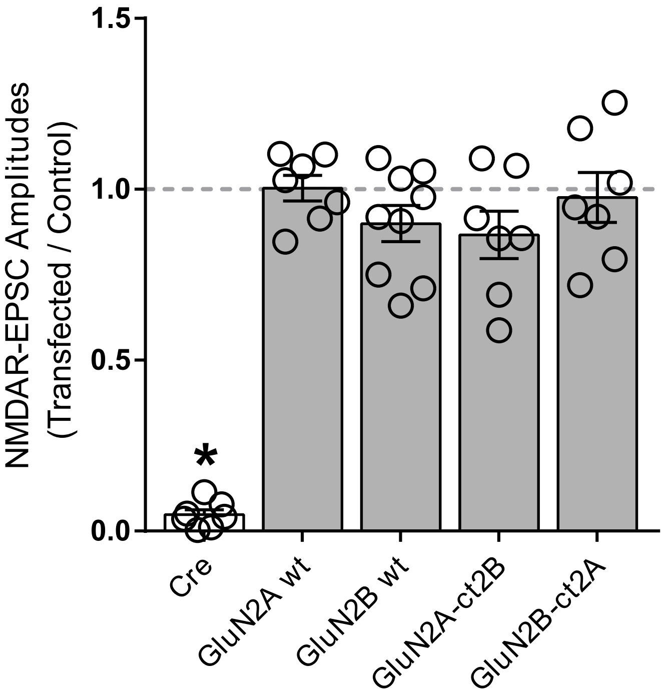

Supplemental Figure S2. Synaptic NMDAR currents from native and chimeric GluN2 constructs. Cultured hippocampal slices were prepared from double-floxed GluN2A/GluN2B (Grin2afl/flGrin2bfl/fl) mice [1, 2] and transfected as described [3] to generate neurons expressing either no GluN2 (Cre), GluN2A only, GluN2B only, chimeric GluN2A containing the GluN2B C-terminal tail, or chimeric GluN2B containing the GluN2A C-terminal tail. NMDA receptor-mediated EPSCs were recorded simultaneously from transfected and adjacent untransfected CA1 pyramidal neurons upon stimulation of Schaffer collaterals. Paired amplitude data were analyzed with a Wilcoxon signed-rank test, and comparisons between groups were performed using a Mann-Whitney U test. Data are means ± s.e.m; * $p < 0.01$ vs no GluN2 (cre transfection only).

1. Akashi, K., T. Kakizaki, H. Kamiya, M. Fukaya, M. Yamasaki, M. Abe, R. Natsume, M. Watanabe, and K. Sakimura, NMDA receptor GluN2B (GluR epsilon 2/NR2B) subunit is crucial for channel function, postsynaptic macromolecular organization, and actin cytoskeleton at hippocampal CA3 synapses. J Neurosci, 2009. **29:**10869-82.
2. Gray, J.A., Y. Shi, H. Usui, M.J. During, K. Sakimura, and R.A. Nicoll, Distinct modes of AMPA receptor suppression at developing synapses by GluN2A and GluN2B: single-cell NMDA receptor subunit deletion in vivo. Neuron, 2011. **71**:1085-101.
3. Schnell, E., M. Sizemore, S. Karimzadegan, L. Chen, D.S. Bredt, and R.A. Nicoll, Direct interactions between PSD-95 and stargazin control synaptic AMPA receptor number. Proc Natl Acad Sci U S A, 2002. **99**:13902-7.

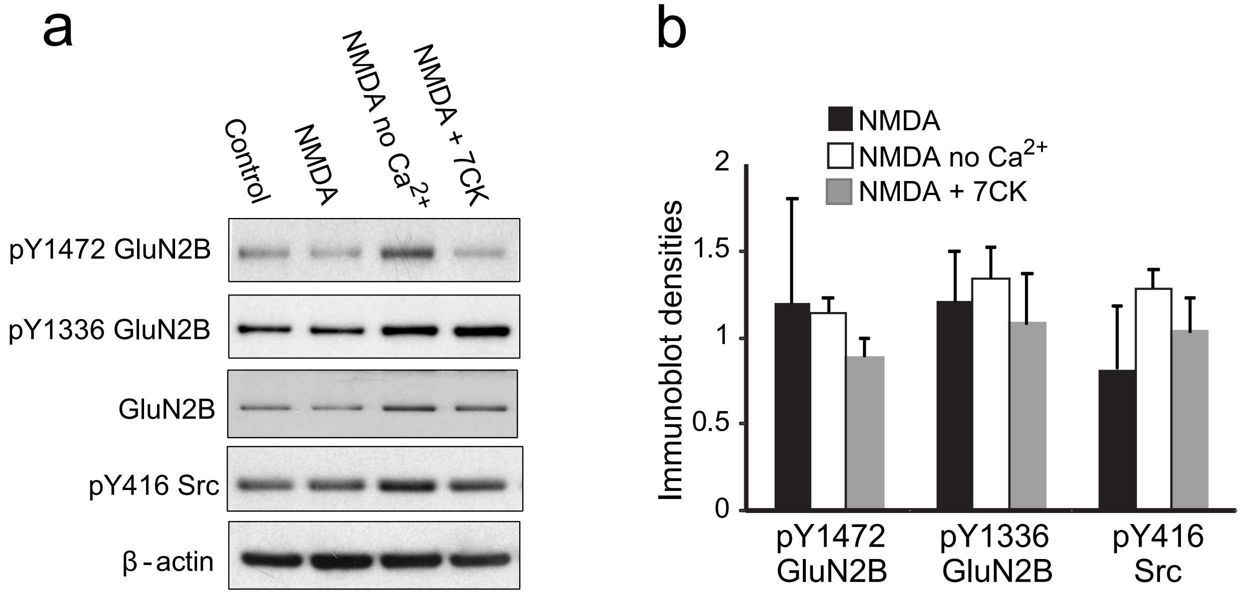

Supplemental Figure S3. Phosphorylation patterns of GluN2B and Src kinases after incubation with NMDA in the presence and absence of 7CK and Ca2+. (**a**) Immunoblots prepared using antibodies that recognize GluN2B phosphorylated at the designated residues and Src kinases phosphorylated at Tyr (Y) 416.
(**b**) Quantification of band densities showed no significant differences among treatment groups. Immunoblot densities of pY1472 GluN2B and pY1336GluN2B are normalized to total GluN2B. Immunoblot density of pY416 Src is normalized to β-actin (n = 3; data are means + s.e.m.).

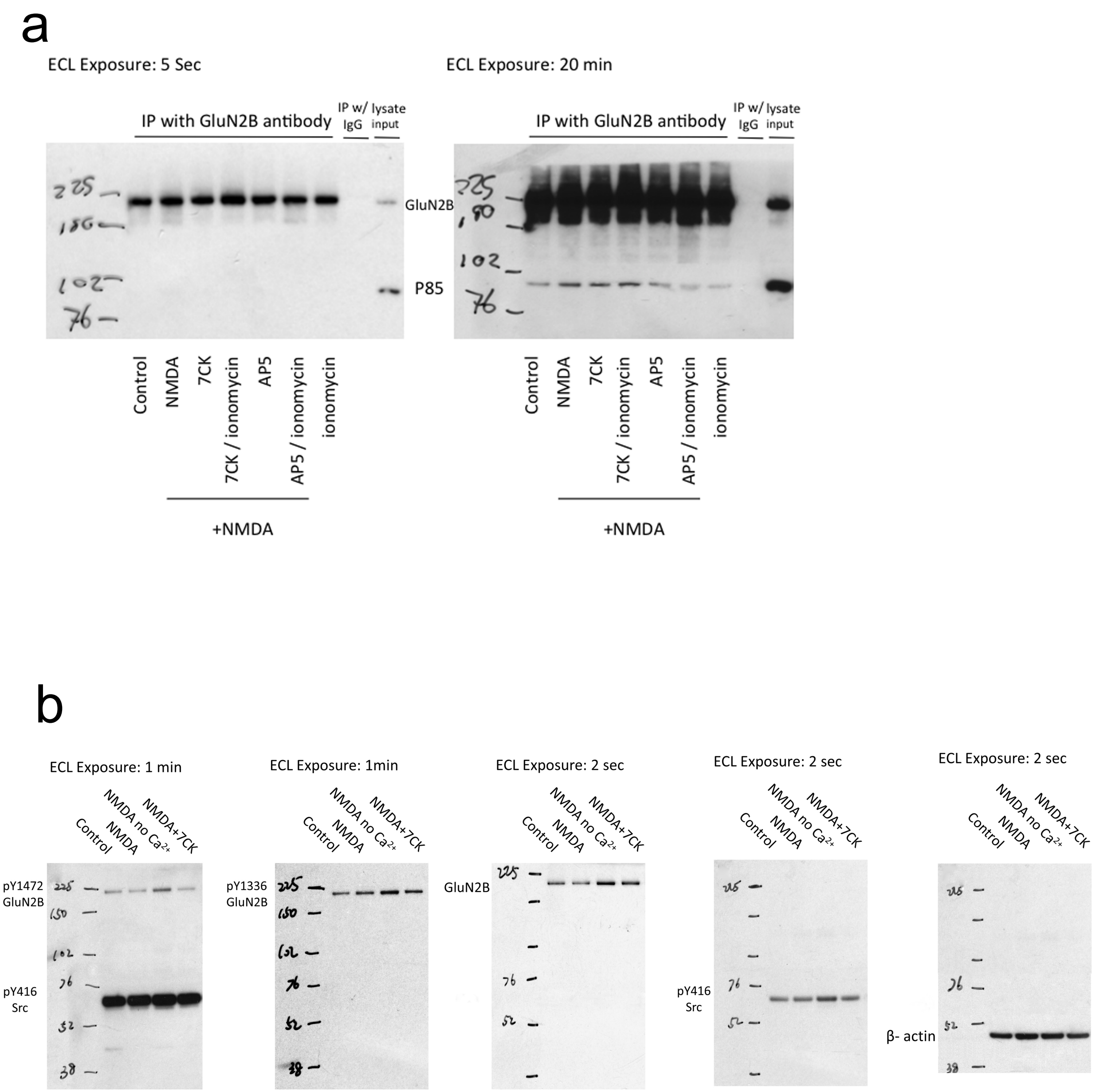

Supplementary Figure S4. Full length blots corresponding to cropped images presented elsewhere. (**a**) Full length blots corresponding to Figure 8c. (**b**) Full length blots corresponding to Supplemental Figure 3a.
